# A systematic review and meta-analysis on how different dexamethasone administration regimes impact total joint arthroplasty outcomes

**DOI:** 10.3389/fphar.2025.1548126

**Published:** 2025-07-03

**Authors:** Can Wang, Chenjing Luo, Xiaoxue Tang, Li Luo, Yuerong Zeng, Yumei Zhang, Xuan Wang

**Affiliations:** ^1^ Department of Pharmacy, The Second Affiliated Hospital of Army Medical University, Chongqing, China; ^2^ Department of Pharmacy, Chongqing General Hospital, Chongqing, China; ^3^ Department of Orthopedics, The Second Affiliated Hospital of Army Medical University, Chongqing, China

**Keywords:** total knee arthroplasty, total hip arthroplasty, dexamethasone, repeated-dose treatment, split-dose treatment, single-dose treatment

## Abstract

**Background:**

Postoperative pain following total joint arthroplasty is a critical factor influencing patient recovery. This meta-analysis evaluated the efficacy and safety of single-dose, repeated-dose, and split-dose perioperative dexamethasone regimens for managing postoperative pain in patients undergoing total joint arthroplasty.

**Methods:**

Randomized controlled studies (RCTs) comparing repeated or split-dexamethasone to single intravenous dexamethasone in patients having total knee/hip arthroplasty were retrieved from Pubmed, the Cochrane Library, Web of Science and Embase databases from inception to October 2024. Using RevMan 5.2, a meta-analysis was performed to evaluate primary outcomes including pain scores, length of stay, and incidence of postoperative rescue analgesia, as well as secondary outcomes such as the incidence of adverse events. Heterogeneity was assessed via I^2^ statistics, and study bias was evaluated using the Cochrane Risk of Bias Assessment Tool.

**Results:**

Twelve trials were included. The results showed that repeated-dose dexamethasone did not differ from single-dose dexamethasone in rest or movement pain scores at 24 h, but significantly reduced both rest (mean difference [MD] = −0.45, 95% confidence interval [CI]: -0.62 to −0.29, *P* < 0.00001, *I* ^*2*^ = 41%) and movement (MD = −0.69, CI: −0.83 to −0.55, *P* < 0.00001, *I* ^*2*^ = 36%) pain scores at 48 h. They also had shorter stays (MD = −0.28, 95% CI: -0.47 to −0.09, *P* = 0.004, *I* ^*2*^ = 71%), lower rates of needing postoperative rescue analgesia (relative risk [RR] = 0.26, 95% CI: 0.11 to 0.63, *P* = 0.003, *I* ^*2*^ = 72%) and postoperative nausea and vomiting [PONV] (RR = 0.47, 95% CI: 0.24 to 0.95, *P* = 0.04, *I* ^*2*^ = 60%). Moreover, patients receiving a single dose of dexamethasone had lower movement scores 24 h postoperatively (MD = 0.26, 95% CI: 0.03 to 0.48, *P* = 0.02, *I* ^*2*^ = 61%) compared to patients with a split-dose of dexamethasone. No significant differences in adverse event rates were observed between single-dose and split-dose dexamethasone.

**Conclusion:**

Compared to patients receiving a single-dose or split-dose of dexamethasone, the administration of repeated doses of dexamethasone can mitigate postoperative pain, reduce the requirement for supplementary opioids, shorten the duration of hospitalization, and decrease the incidence of PONV following arthroplasty.

**Systematic Review Registration:**

https://inplasy.com/inplasy-2023-10-0023/.

## Introduction

Total knee and hip arthroplasty (TKA and THA) are efficacious interventions for managing advanced osteoarthritis, rheumatoid arthritis, post-traumatic arthritis, and other end-stage degenerative disorders of the knee and hip (Konnyu et al., 2023; Zhang et al., 2024). However, surgical trauma and primary joint pathology in these procedures can elicit robust pain responses, leading to intense postoperative pain (Summers et al., 2020). Inadequate management of postoperative pain has been shown to elevate the likelihood of developing chronic pain, impede patient recovery, extend hospitalization duration, and exacerbate financial burdens (Aroke et al., 2020). Postoperative nausea and vomiting (PONV) is a prevalent issue, with an incidence rate of 20%–40%, which might compromise patients’ post-surgical recovery and diminish their overall satisfaction with the surgical procedure (Yang et al., 2025). Hence, the primary considerations for facilitating patients’ postoperative recovery involve alleviating postoperative pain and preventing PONV.

Dexamethasone is a synthetic glucocorticoid with prolonged activity and several therapeutic benefits, including anti-inflammatory, anti-allergic, immunosuppressive, and anti-shock properties (Chan et al., 2020). Consequently, it has been used as a multimodal analgesic approach following joint arthroplasty surgery (Joshi, 2023; Wainwright et al., 2020). Previous studies have demonstrated that intravenous (IV) administration of dexamethasone during perioperative period can effectively mitigate postoperative pain, prevent PONV, and reduce the need for opioid medications in patients undergoing joint replacement procedures (Fan et al., 2018; Liang et al., 2022). Nevertheless, there remains a lack of clarity regarding the most effective dosage and administration route of dexamethasone during the perioperative phase of joint replacement procedures. Based on current evidence, there are mainly three regimens for perioperative IV administration of dexamethasone: the single-dose regimen (single preoperative IV administration of a fixed dose, such as 10 mg), the repeated-dose regimen (preoperative IV administration of 10 mg followed by a 10-mg repeat dose postoperatively), and the split-dose regimen (where the total 10-mg dose is divided equally into 5 mg IV before surgery and 5 mg IV after surgery) (Backes et al., 2013; Chen et al., 2024). The safety of dexamethasone is questionable due to concerns regarding its potential adverse effects on delayed incision healing, infection, and hyperglycemia.

The current meta-analysis investigates the impact of three distinct dosing regimens - a single dose, repeated doses, and split doses of dexamethasone - during the perioperative phase of joint arthroplasty on postoperative pain, as well as their impact on length of stay, incidence of postoperative remedial analgesia, and incidence of adverse events.

## Methods

The meta-analysis used the procedures outlined in the Preferred Reporting Items for Systematic Reviews and Meta-Analyses (PRISMA) (Page et al., 2021). Protocol registration was done at INPLASY with the identification number INPLASY2023100023.

### Search strategy

PubMed, the Cochrane Library, Web of Science and Embase databases were comprehensively searched (from inception to October 2024). The search query included (dexamethasone) AND (total knee arthroplasty OR total knee replacement OR TKA OR TKR OR total hip arthroplasty OR total hip replacement OR THA OR THR OR total joint arthroplasty OR total joint replacement OR TJA OR TJR). Two researchers conducted separate screenings to find and select papers satisfying the predetermined inclusion and exclusion criteria. Additionally, the references within each retrieved publication were examined to discover any potentially overlooked relevant investigations. In cases of differences, the study group negotiated to reach a consensus.

### Inclusion criteria

The researchers employed the following criteria to include relevant investigations: (1) individuals undergoing primary unilateral THA/TKA, (2) Randomized Controlled Trials (RCTs) that compared repeated or split doses to a single dose of intravenous dexamethasone for pain relief, and (3) the outcomes of interest in these studies included postoperative pain scores, the number of patients requiring additional pain medication after surgery, the length of hospital stay (LOS), PONV incidence, and any adverse reactions related to dexamethasone such as infection, hyperglycemia, and gastrointestinal bleeding. Exclusion criteria were as follows: (1) RCTs with incomplete outcome data that could not be supplemented by contacting authors; (2) studies conducted in pediatric populations or patients with severe systemic diseases (e.g., uncontrolled diabetes, active infection) that might confound the effects of dexamethasone; (3) non-English publications.

The studies were identified by two separate reviewers who used EndNote X9 to screen titles and abstracts. After this initial screening, the same reviewers conducted a full-text evaluation of the shortlisted studies. Any discrepancies was resolved through discussion with a third reviewer.

### Data extraction

Two investigators independently conducted data extraction from selected studies, and the extracted variables were categorized as follows: (1) study characteristics including the primary author’s name, publication year, surgical procedure type, sample size, and anesthesia technique; (2) intervention details covering the time and dosage of dexamethasone administration, comparison regimen, and postoperative pain management protocol; (3) outcome measures such as postoperative pain scores (VAS or other scales), length of hospital stay, additional analgesic use, and adverse events, including PONV, surgical site infections, gastrointestinal bleeding, and venous thromboembolism (VTE).

Ranging from 0 to 100 mm, the visual analog scale (VAS) was transformed into a 10-point VAS. Multiple studies (Shafshak and Elnemr, 2021; Thong et al., 2018) have demonstrated a high correlation between VAS and NRS, with strong agreement between the two scales as shown by statistical analyses. Therefore, pain scores from studies using the Numerical Rating Scale (NRS) were converted to 10-point VAS equivalents. The necessary data were extracted and standardized using the official Cochrane data conversion tool and the graphical data extraction application Web Plot Digitizer.

### Quality evaluation

Two independent researchers evaluated the risk of bias in included studies using the Cochrane Risk of Bias Assessment Tool, which assesses studies across seven domains: random sequence generation, allocation concealment, blinding of participants and personnel, blinding of outcome assessment, incomplete outcome data, selective reporting, and other bias. They categorized studies as “low risk,” “high risk,” or “unclear” for items under consideration (Sterne et al., 2019). If a study met the low - risk criteria in all key domains, it was considered to have a low risk of bias; if it had high - risk issues in one or more key domains, it was rated as having a high risk of bias; and if there was insufficient information to determine the risk level in one or more domains or some domains had unknown risks, the study was classified as having an unclear risk of bias. All conflicts were successfully resolved through the process of negotiation.

### Data analysis

All statistical analyses were conducted using Review Manager (RevMan version 5.2, Cochrane Community, London, England) software. If the study reported data using the median and interquartile range or p-value and confidence interval, we transformed these into means and standard deviations using methods described by Luo (Luo et al., 2018), Wan (Wan et al., 2014), and Cochrane calculator. In cases where there were multiple subjects in the experimental group, to prevent the repeated utilization of the control group’s sample size, it was evenly divided based on the methodology reported in prior research (Cumpston et al., 2019).

Statistical heterogeneity was assessed using *I* ^*2*^, and the selection of effect models was based on the outcomes of this assessment. When the p-value was less than 0.10 or *I* ^*2*^ was greater than 50%, random-effect models were employed. Continuous variables were assessed using weighted mean differences (WMD) and 95% confidence intervals (CI). The assessment of dichotomous variables involved the utilization of relative risk ratios (RR) and 95% CIs. The findings are presented in forest plots, and *P* < 0.05 was considered statistically significant. When the number of included studies exceeded 10, funnel plots combined with Egger’s test were used for publication bias evaluation; for datasets with ≤10 studies, publication bias assessment was deferred due to insufficient statistical power, and sensitivity analyses were performed to evaluate result robustness.

## Results

### Search results

**FIGURE 1:**
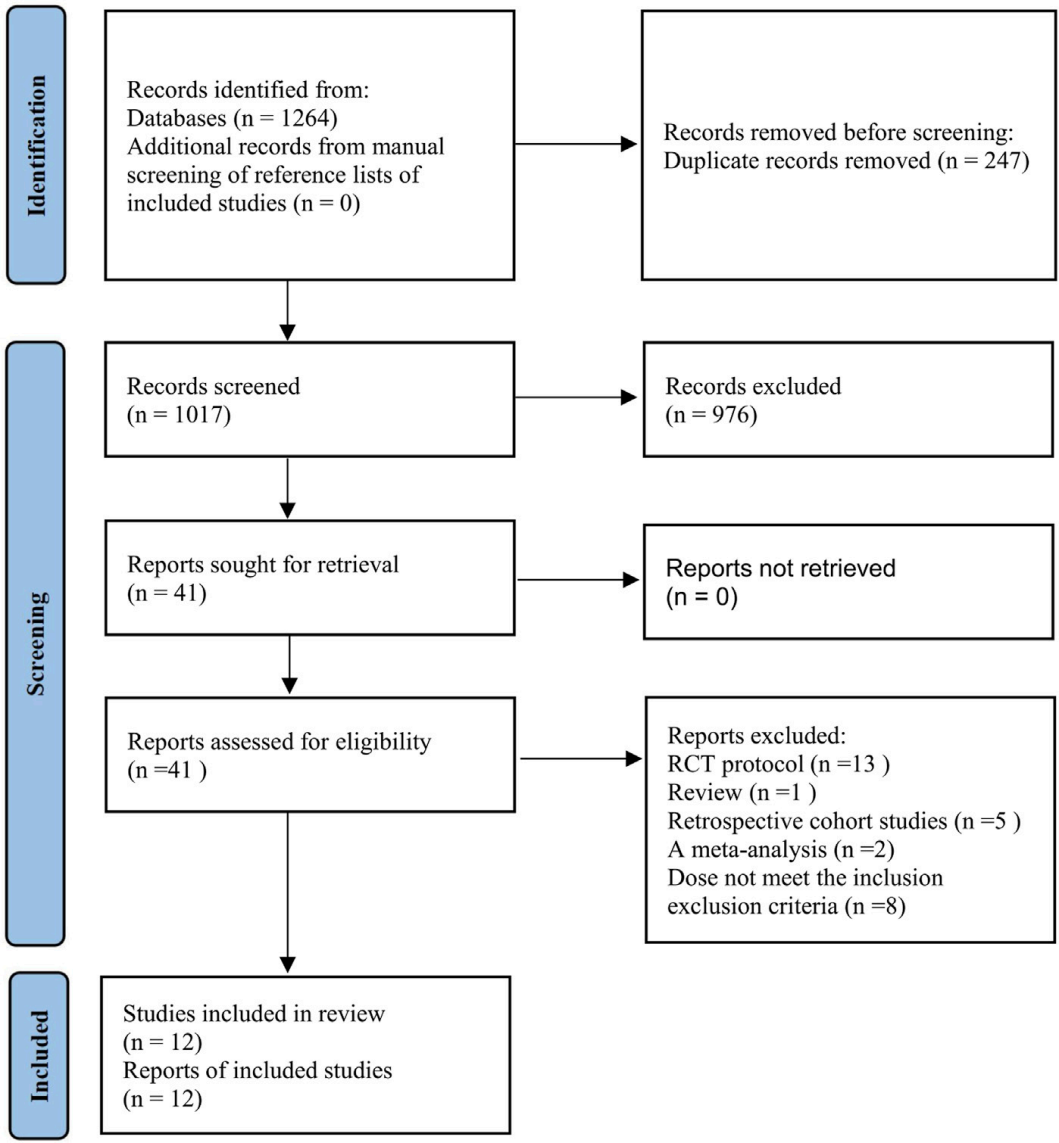
Flow diagram of the study selection process and results.

The PRISMA flow chart depicts the comprehensive search and screening of the existing literature, the method of identifying relevant sources, and the rationale for excluding certain studies. In the final analysis, twelve studies (Backes et al., 2013; Chen et al., 2024; Gasbjerg et al., 2022; Lei et al., 2022; Lei et al., 2020a; Lei et al., 2020b; Liu et al., 2024; Lucero et al., 2021; Saini et al., 2023; Wu et al., 2018; Xie et al., 2024; Xu et al., 2018) were included (Figure 1).

Among the included trials, three studies (Lei et al., 2020a; Lei et al., 2020b; Lucero et al., 2021) exclusively focused on patients who underwent THA, while another six studies (Chen et al., 2024; Gasbjerg et al., 2022; Lei et al., 2022; Saini et al., 2023; Wu et al., 2018; Xu et al., 2018) focused on patients who underwent TKA, and three studies (Backes et al., 2013; Liu et al., 2024; Xie et al., 2024) included both THA and TKA patients. Notably, all participants in the included studies underwent surgery under either general anesthesia or spinal anesthesia. Within the studies analyzed, the administered doses of dexamethasone varied from 4 to 24 mg for single administrations. Additionally, seven studies (Backes et al., 2013; Gasbjerg et al., 2022; Lei et al., 2020a; Lucero et al., 2021; Wu et al., 2018; Xie et al., 2024; Xu et al., 2018) incorporated repeated doses of dexamethasone, while five studies (Chen et al., 2024; Lei et al., 2022; Lei et al., 2020b; Liu et al., 2024; Saini et al., 2023) contained split doses of the medication. Table 1 displays the baseline characteristics, as well as the specifics of the interventions.

**TABLE 1:** Characteristics of included studies.

Author, year	Surgical type	Anesthesia type	Intervention (n)	Dexamethasone dose administration	Primary Outcome(s)
Lei et al. (2020a)	THA	General	E1:10 mg IV DEX (50) E2:10 mg + 10 mg IV DEX (50) E3:10 mg + 10 mg + 10 mg IV DEX (50)	E1: Preop E2: Preop, post-24 h E3: Preop, post-24h, post-48 h	Pain score
Lei et al. (2020b)	THA	General	E1:20 mg IV DEX (55) E2:10 mg + 10 mg IV DEX (55) C:Equal volume of NS (55)	E1: Preop E2: Preop, post-24 h	LOS, Pain score
Lucero et al. (2021)	THA	Spinal	E1:8 mg IV DEX (54) E2:8 mg + 8 mg IV DEX (1)	E1: Preop E2: Preop, post-8 h	LOS, Pain score
Backes et al. (2013)	THA/TKA	General	E1: 10 mg IV DEX (41) E2: 10 mg + 10 mg IV DEX (42)	E1: Preop E2: Preop, post-24 h	LOS, Pain score, Opioid consumption
Liu et al. (2024)	THA/TKA	General	E1:20 mg IV DEX (46) E2:10 mg + 10 mg IV DEX (45) C:Equal volume of NS(45)	E1: Preop E2: Preop, post-24 h	LOS, Pain score
Xie et al. (2024)	THA/TKA	General	DX1: 10 mg IV DEX (108) DX2: 10 mg + 10 mg + 10 mg IV DEX (116) C:Equal volume of NS (108)	E1: Preop E2: Preop, post-6 h, post-24 h	LOS
Wu et al. (2018)	TKA	General	E1:10 mg IV DEX (50) E2: 10 mg + 10 mg IV DEX (50) C:2 mL NS (50)	E1: Preop E2: Preop, post-6 h	LOS, Pain score
Xu et al. (2018)	TKA	General	E1: 20 mg IV DEX (60) E2: 20 mg + 10 mg + 10 mg IV DEX (61) C: Equal volume of NS (61)	E1: Preop E2: Preop, post-24 h, post-48 h	LOS, Pain score
Lex et al. (2021)	TKA	General	E1: 20 mg IV DEX (62) E2: 10 mg + 10 mg IV DEX (67) C:Equal volume of NS (63)	E1: Preop E2: Preop, post-24 h	Pain score
Gasbjerg et al. (2022)	TKA	Spinal or general	E1: 24 mg IV DEX (161) E2: 24 mg + 24 mg IV DEX (162) C:6 mL NS (162)	E1: Preop E2: Preop, post-24 h	Pain score, Opioid consumption
Saini et al. (2023)	TKA	Spinal	E1:8 mg periarticular injection DEX (60) E2: 8 mg IV DEX (60) E3:4 mg + 4 mg IV DEX (60)	E1: Preop E2: Preop E3: Preop, post-24 h	Opioid consumption
Chen et al. (2024)	TKA	General	E1: 10 mg IV DEX (50) E2: 5 mg + 5 mg IV DEX (50) C:Equal volume of NS (50)	E1: Preop E2: Preop, returned to the ward	Pain score

E1, Experimental Group 1; E1, Experimental Group 1; E2, Experimental Group 2; C, control group; DEX:dexamethasone; NS, normal saline; Preop, preoperative; post-24 h, postoperative 24 h; post-48 h, postoperative 48 h; post-6 h, postoperative 6 h; LOS, length of stay.

### Literature quality evaluation

**FIGURE 2:**
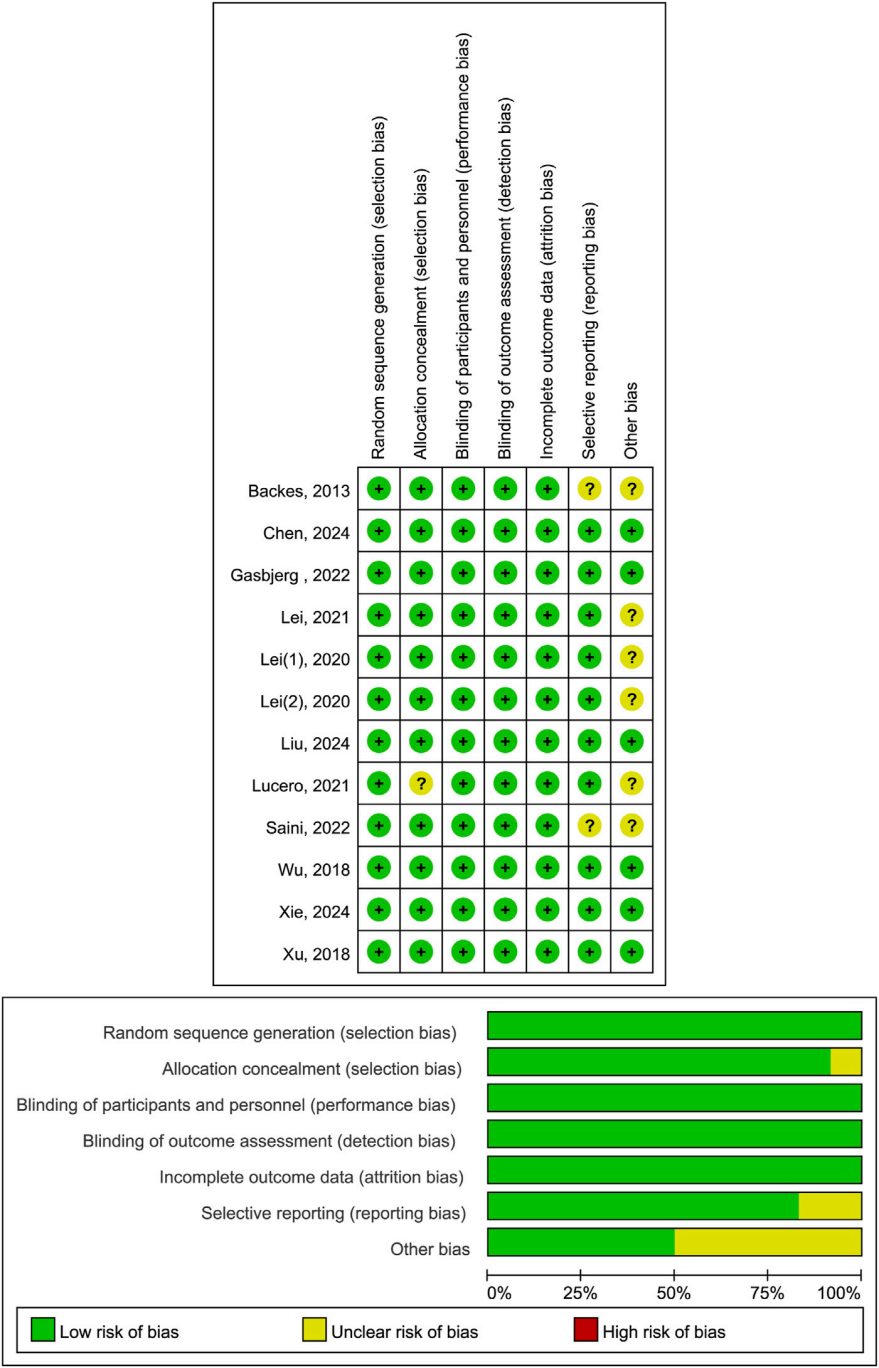
Risk of bias summary.

Trial quality assessment revealed that six studies (Chen et al., 2024; Gasbjerg et al., 2022; Liu et al., 2024; Wu et al., 2018; Xie et al., 2024; Xu et al., 2018) had a low risk of bias, while another six (Backes et al., 2013; Lei et al., 2022; Lei et al., 2020a; Lei et al., 2020b; Lucero et al., 2021; Saini et al., 2023) had a moderate risk of bias. The primary factors contributing to this moderate risk were inadequate allocation concealment, selective reporting of research results, and uncertainty regarding other potential sources of bias (Figure 2).

### Data synthesis

#### Pain scores

**FIGURE 3:**
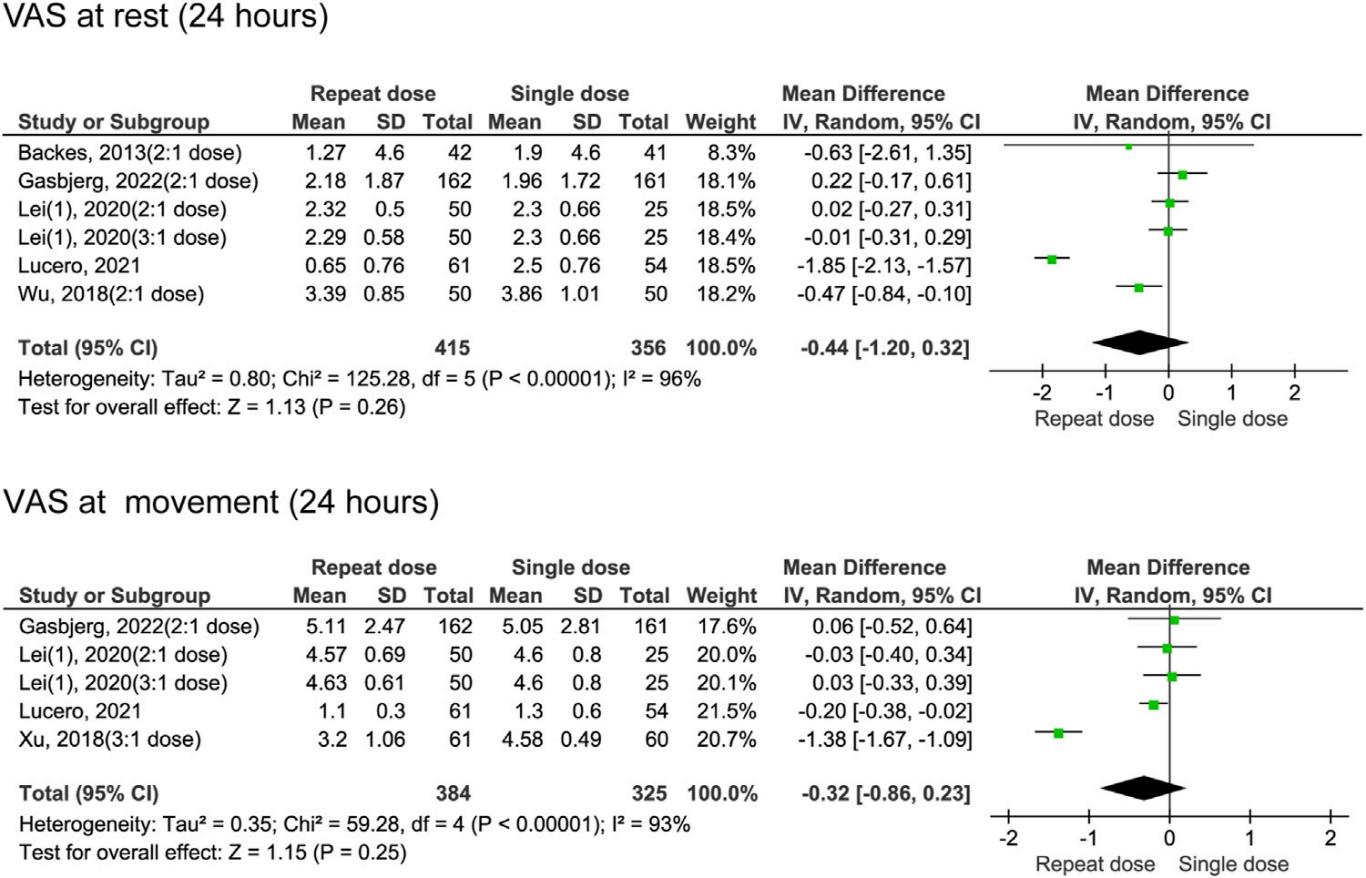
The 24-h VAS pain scores between the repeated and single-dose dexamethasone groups (Forest plot).

**FIGURE 4:**
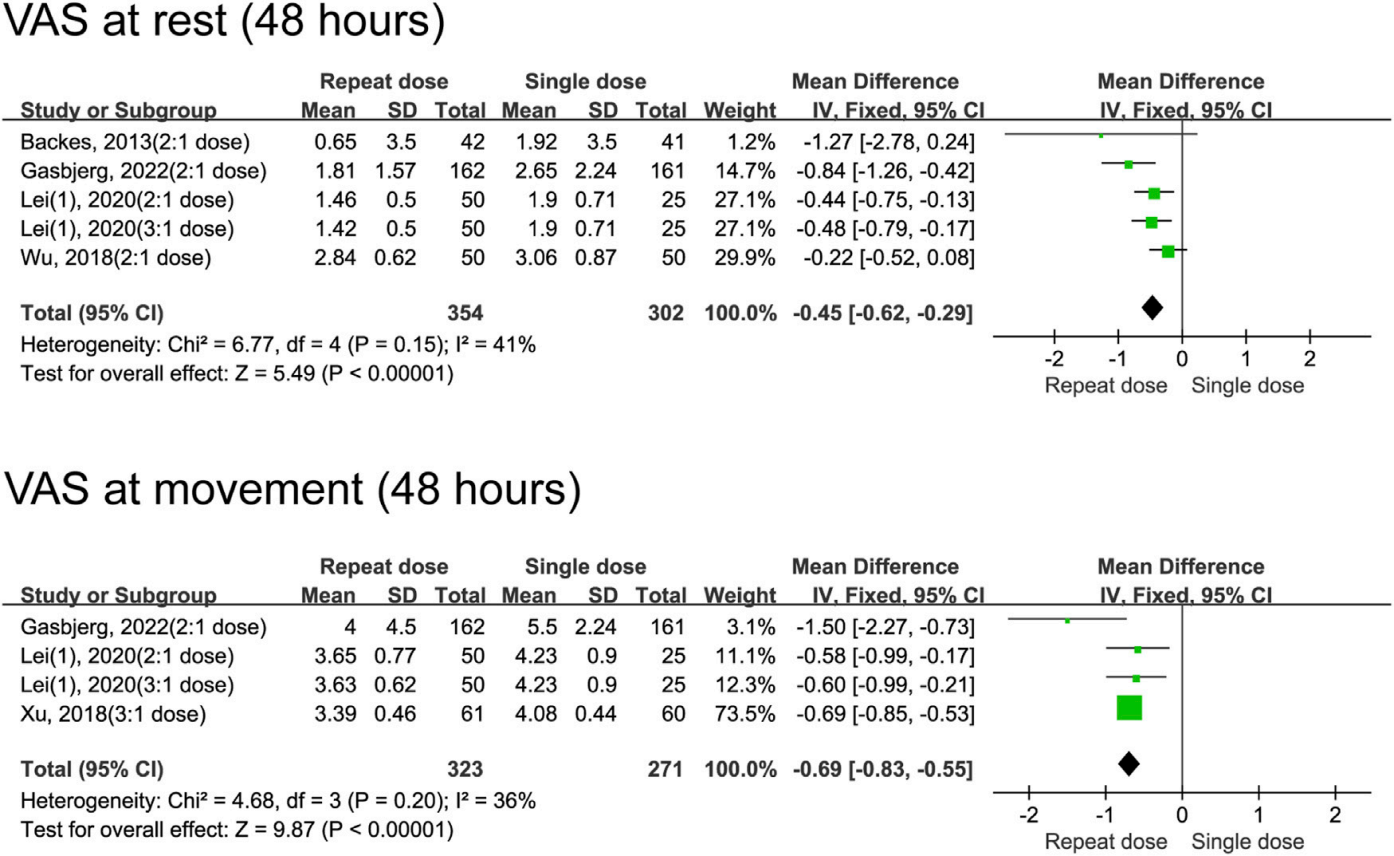
The 48-h VAS pain scores between the repeated and single-dose dexamethasone groups (Forest plot).

At 24 h following surgery, pain scores at rest (MD = −0.44, 95% CI: −1.20 to 0.32, *P* = 0.26, *I* ^*2*^ = 96%) and during movement (MD = −0.32, 95% CI: −0.86 to 0.23, *P* = 0.25, *I* ^*2*^ = 93%) were not different between single and repeated dexamethasone regimens (Figure 3). High heterogeneity (96% and 93%, respectively) indicates major differences between the studies in terms of populations, interventions, or measurements. However, at 48 h after the surgery, the group receiving repeated doses of dexamethasone exhibited lower rest (MD = −0.45, 95% CI: −0.62 to −0.29, *P* < 0.00001, *I* ^*2*^ = 41%) and movement (MD = −0.69, 95% CI: -0.83 to −0.55, *P* < 0.00001, *I* ^*2*^ = 36%) pain scores (Figure 4).

**FIGURE 5:**
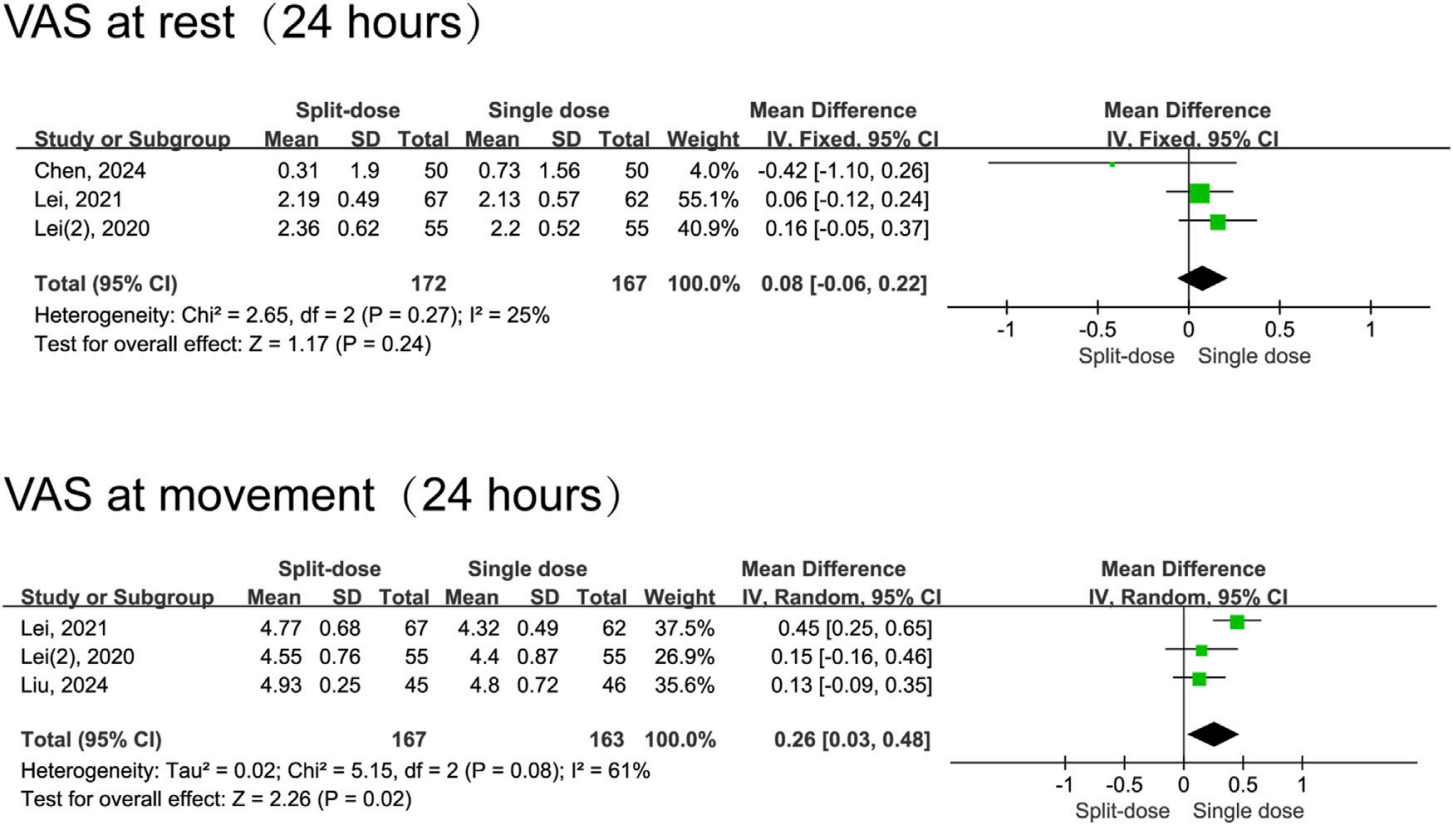
The 24-h VAS pain scores between the split-dose and single-dose dexamethasone groups (Forest plot).

**FIGURE 6:**
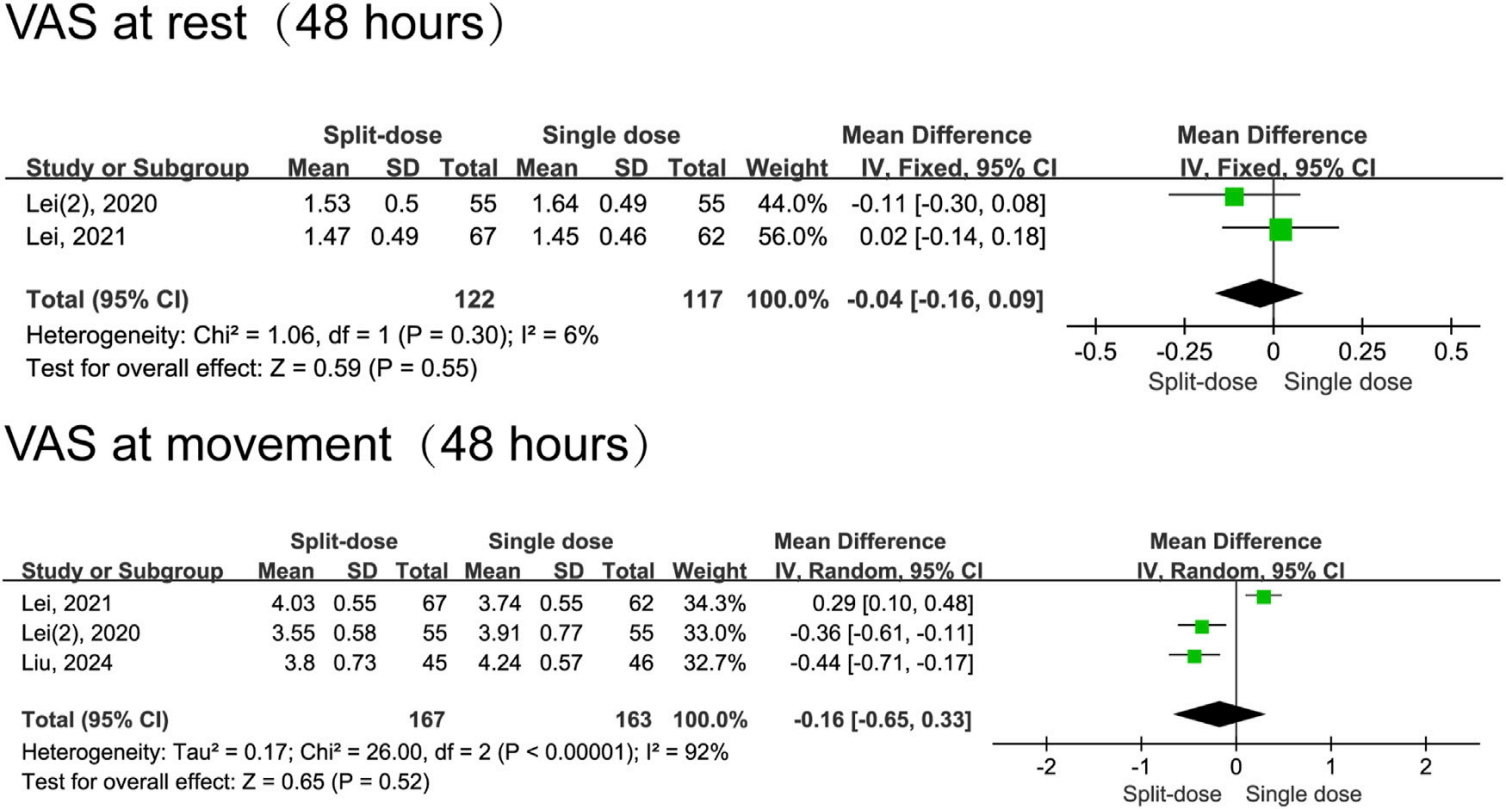
The 48-h VAS pain scores between the split-dose and single-dose dexamethasone groups (Forest plot).

In comparison to split-dose dexamethasone, the administration of a single dose of dexamethasone resulted in a reduction in pain scores during movement 1 day following surgery (MD = 0.26, 95% CI: 0.03 to 0.48, *P* = 0.02, *I* ^*2*^ = 61%). Notably, this analysis included only three studies, and moderate heterogeneity was observed for 24-h movement pain scores. Nevertheless, the two groups had comparable resting pain scores at 24 h (MD = 0.08, 95% CI: -0.06 to 0.22, *P* = 0.24, I^2^ = 25%) and 48 h postoperatively (MD = −0.04, 95% CI: −0.16 to 0.09, *P* = 0.55, I^2^ = 6%), as well as in movement pain scores at 48 h (MD = −0.16, 95% CI: −0.65 to 0.33, *P* = 0.52, I^2^ = 92%) (Figures 5, 6).

#### Length of stay

**FIGURE 7:**
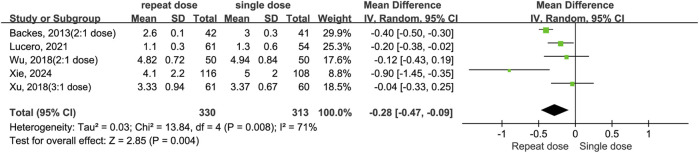
The length of hospital stay between the repeated and single-dose dexamethasone groups (Forest plot).

Five studies (Backes et al., 2013; Lucero et al., 2021; Wu et al., 2018; Xie et al., 2024; Xu et al., 2018) examined the duration of hospitalization following surgery. Data shows repeated administrations of dexamethasone decreased LOS when compare to the single-dose regimen (MD = −0.28, 95% CI: −0.47 to −0.09, *P* = 0.004). However, the high heterogeneity observed (*I* ^*2*^ = 71%, Chi^2^ = 13.84, *P* = 0.008) implies results were not consistent across studies (Figure 7).

#### Incidence of postoperative remedial analgesia

**FIGURE 8:**
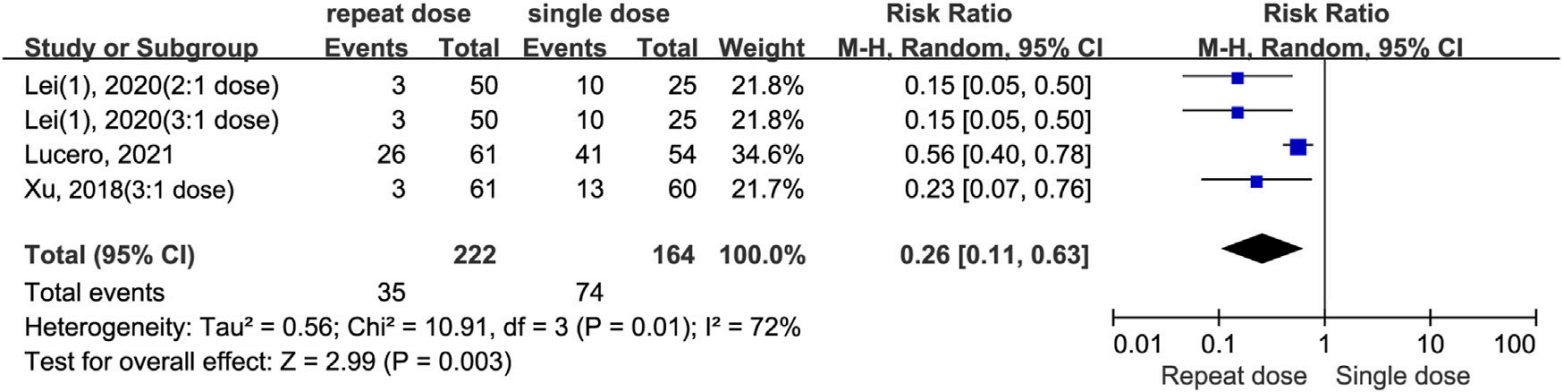
The incidence of postoperative remedial analgesia between the repeated and single-dose dexamethasone groups (Forest plot).

**FIGURE 9:**
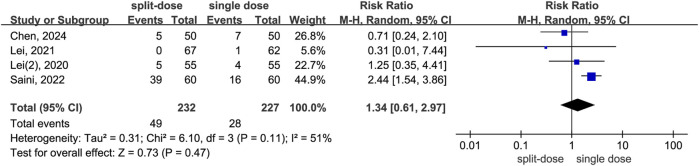
The incidence of postoperative remedial analgesia between the split-dose and single-dose dexamethasone groups (Forest plot).

The meta-analysis shows repeat dosing of dexamethasone significantly reduces the risk for postopetative remedial analgesia compared to a single-dose regimen (RR = 0.26, 95% CI: 0.11 to 0.63, *P* = 0.003, *I* ^*2*^ = 72%). Despite moderate-to-high heterogeneity (*I* ^*2*^ = 72%, Chi^2^ = 10.91, *P* = 0.01) and variance in effect sizes (Tau^2^ = 0.56), all studies individually and as a whole favour the repeat-dose strategy (Figure 8). By contrast, the split-dose group had a 34% higher risk of needing postoperative remedial analgesia compared to the single-dose group, although this difference was not statistically significant (RR = 1.34, 95% CI: 0.61 to 2.97, *P* = 0.47, *I* ^*2*^ = 51%) (Figure 9).

#### Incidence of adverse events

**FIGURE 10:**
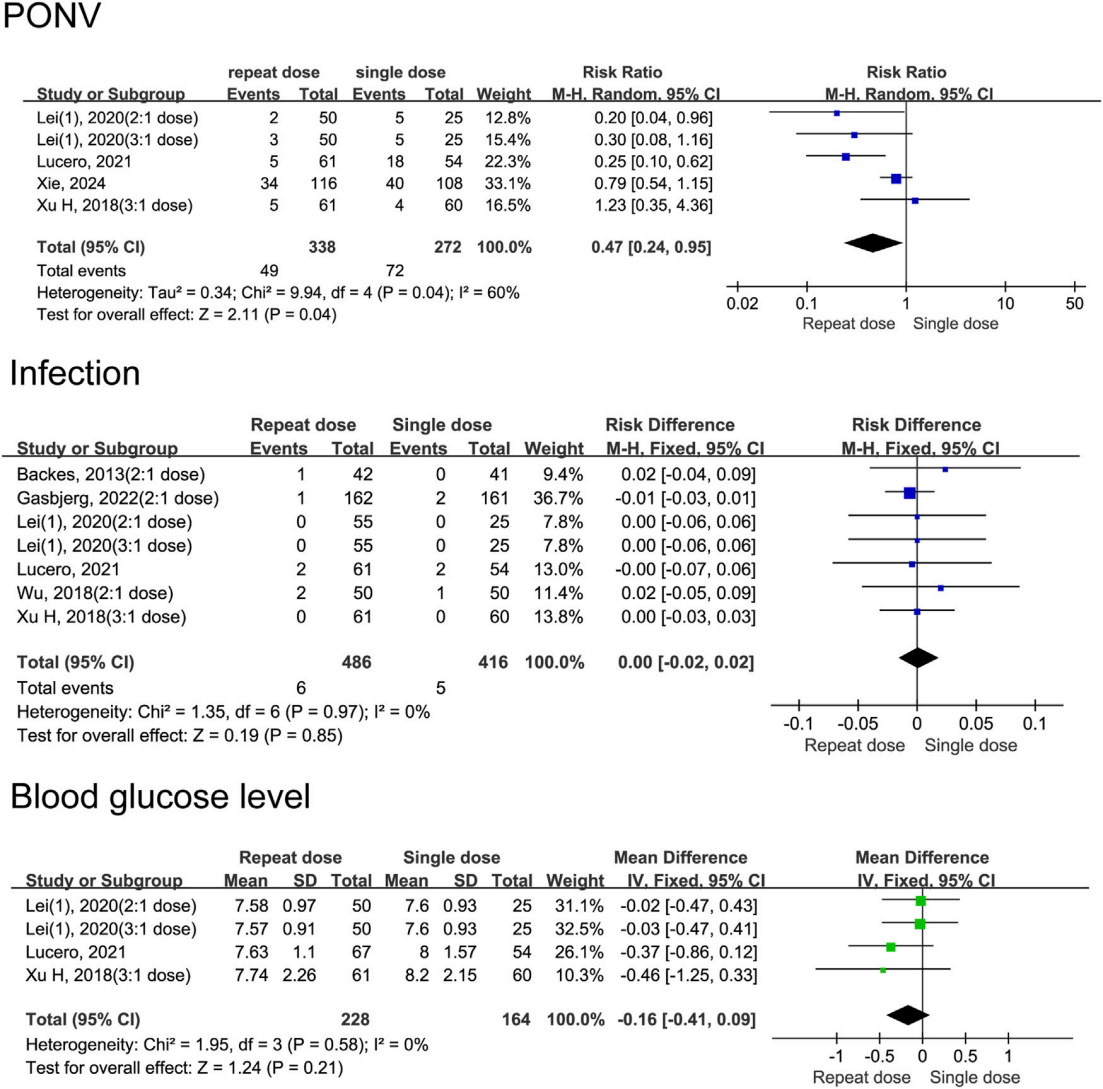
Adverse event rates between the repeated and single-dose dexamethasone groups (Forest plot).

**FIGURE 11:**
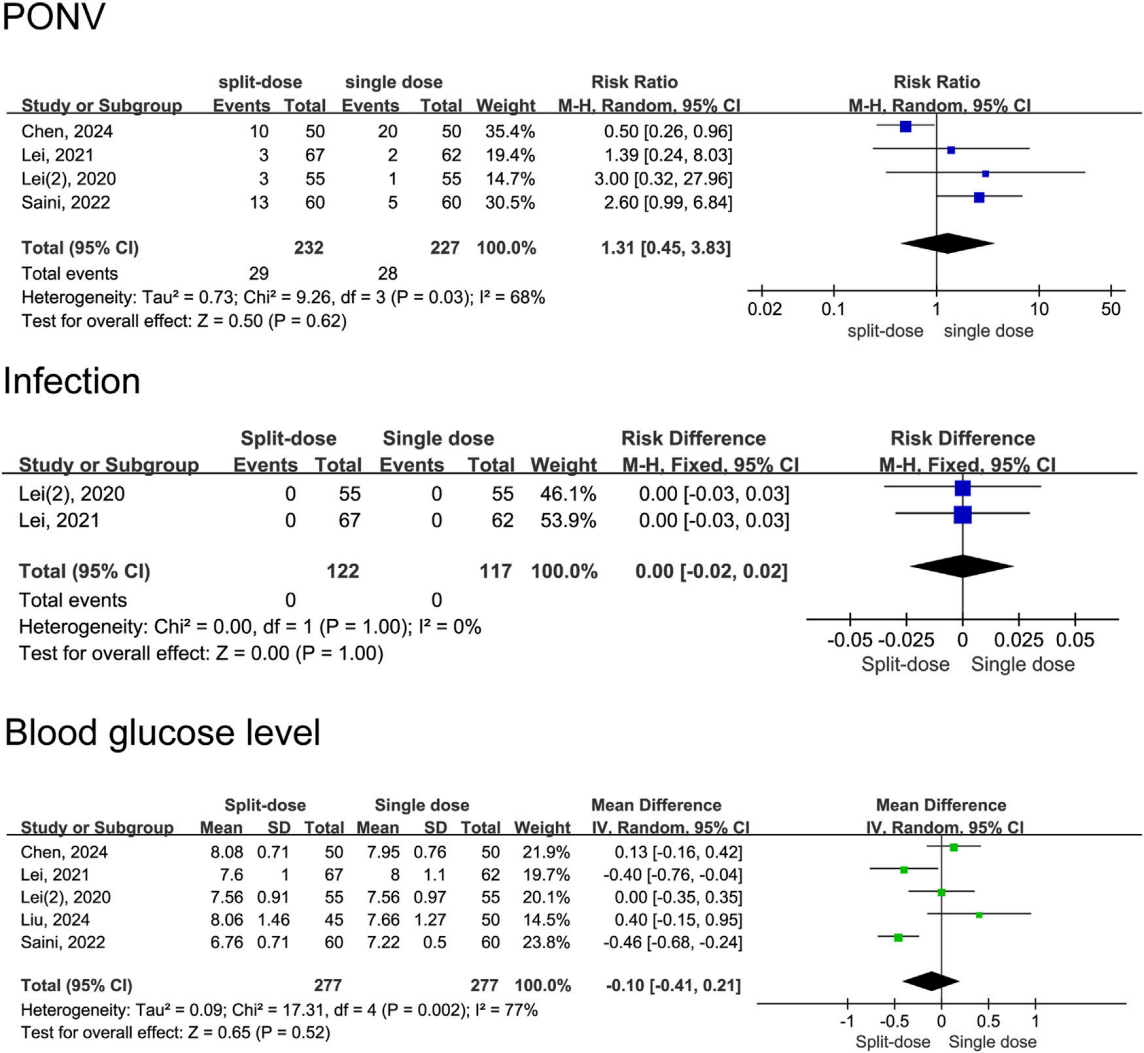
Adverse event rates between the split-dose and the single-dose dexamethasone groups (Forest plot).

Our data indicate that patients receiving repeat-dose dexamethasone had 53% lower risk of experiencing PONV than those receiving a single dose (RD = 0.47, 95% CI: 0.24 to 0.95, *P* = 0.04). However heterogeneity is moderate (*I* ^*2*^ = 60%, Chi^2^ = 9.94, *P* = 0.04), indicating some variability among studies. Data shows no significant difference in infection risk between patients receiving repeat-dose versus single-dose dexamethasone (RD = 0.00, 95% CI: −0.02 to 0.02, *P* = 0.85) with high consistency among the included studies (*I* ^*2*^ = 0%, Chi^2^ = 1.35, *P* = 0.97). There is no significant difference in blood glucose levels between patients receiving repeat-dose versus single-dose dexamethasone (MD = −0.16, 95% CI: −0.41 to 0.09, *P* = 0. 21), although the mean difference slightly favours repeat dosing. Consistency is high among the included studies (*I* ^*2*^ = 0%, Chi^2^ = 1.95, *P* = 0.58) (Figure 10). In the group of patients who received split-dose dexamethasone, there were no significant differences incidence of PONV (RR = 1.31, 95% CI: 0.45 to 3.83, *P* = 0.62, *I* ^*2*^ = 68%) and blood glucose levels (MD = −0.10, 95% CI: −0.41 to 0.21, *P* = 0.52, *I* ^*2*^ = 77%) compared to the group that received single-dose dexamethasone (Figure 11). Two studies (Lex et al., 2021; Lei et al., 2020a) reported data on infection incidence, with both groups showing zero event rates. Gastrointestinal bleeding events were not documented in any of the trials included. Only one study (Gasbjerg et al., 2022) provided data on VTE events, reporting 1 case of thrombosis in the single-dose group and 3 cases in the repeat-dose group. However, the difference in incidence did not reach statistical significance.

### Sensitivity analysis

To evaluate the robustness of the meta - analysis results, sensitivity analyses were conducted using leave - one - out methods. The sensitivity analysis showed that omitting any single study did not excessively influence the results.

## Discussion

There is an ongoing debate on the optimal pain management approach for patients following total joint arthroplasty (Anger et al., 2021; Lavand’homme et al., 2022). Our study is a comprehensive analysis encompassing twelve RCTs to investigate the impacts of various dexamethasone delivery regimens in the context of total joint arthroplasty. This research aimed to enhance the knowledge of orthopedic surgeons and patients on dexamethasone, ultimately leading to improved decision-making efficiency in selecting perioperative drugs.

Multimodal analgesia has become the standard for postoperative pain management in total joint arthroplasty, combining analgesics with distinct mechanisms to enhance efficacy and reduce side effects. This approach typically includes nonsteroidal anti-inflammatory drugs (NSAIDs), opioids, acetaminophen, and glucocorticoids like dexamethasone (Karam et al., 2021), targeting multiple pain pathways to minimize single-drug reliance. Dexamethasone stands out in this paradigm due to its unique long-acting anti-inflammatory profile. Unlike short-acting NSAIDs, dexamethasone exerts prolonged effects by inhibiting phospholipase A_2_, thereby reducing prostaglandin and leukotriene synthesis (Steiness et al., 2024). This mechanism allows dexamethasone to suppress deep-tissue inflammation and pain sensitization, complementing the rapid-onset but short-lived action of NSAIDs. Prior meta-analyses have provided evidence supporting dexamethasone effectiveness in lowering postoperative pain scores, diminishing LOS and diminishing postoperative complications (Fan et al., 2018; Liang et al., 2022; Hannon et al., 2022). Consequently, dexamethasone has been a commonly employed intervention during the perioperative phase of total joint arthroplasty (Li et al., 2018; Meng and Li, 2017; Zhou et al., 2018). Notably, our research diverges from earlier findings (Liang et al., 2022): repeated dexamethasone dosing after arthroplasty achieved significantly greater reduction in 48-h VAS pain scores compared with single-dose administration, whereas no significant difference was observed at 24 h. This finding aligns with Danish investigators’ (Nielsen et al., 2022) report in high-pain-response TKA patients: dexamethasone induced a 40% pain increase at 48 h post-surgery, with levels further escalating to 53% above baseline in the subsequent period. Pharmacodynamic analysis confirmed dexamethasone’s anti-inflammatory effect dissipated at 48 h, directly leading to pain rebound. These converging results underscore that repeating dexamethasone on Day 1 or 2 postoperatively may counteract the 48-h efficacy decay, thereby prolonging pain control and suppressing the surgical inflammatory response.

The evaluation of analgesic efficacy also includes considering the requirement for remedial analgesic medication. Our investigation revealed a noteworthy discrepancy among cases necessitating rescue analgesia within the repeat-dose dexamethasone group and the single-dose dexamethasone group, with the former exhibiting a much lower proportion. The observation above is corroborated by a recent investigation that examined the impact of administering dexamethasone in repeated doses compared to a single dose and determined that repeated perioperative doses of dexamethasone resulted in a decrease in perioperative pain and opiate usage during hospitalization (Arraut et al., 2023).

Postoperative PONV is a significant variable that impacts the recovery of patients. The prevention of PONV can expedite the postoperative recovery process and reduce the duration of hospitalization. Our findings indicate that PONV occurrence was lower in the repeat-dose dexamethasone group than in the single-dose dexamethasone group. Additionally, repeat-dose dexamethasone had a greater impact on reducing the length of postoperative hospital stay compared to single-dose dexamethasone, which aligns with prior meta-analyses (Lex et al., 2021). However, our research also revealed a comparable occurrence of postoperative PONV between the single-dose dexamethasone group and the split-dose dexamethasone group. Consequently, more comparative studies are required to ascertain the optimal dosage and timing schedule for dexamethasone administration.

Dexamethasone use should be carefully considered due to its association with potential complications such as impaired wound healing, infection, and hyperglycemia. However, according to a meta-analysis’s findings, perioperative corticosteroids in individuals undergoing total joint arthroplasty were not linked to a heightened susceptibility to postoperative infections (Feeley et al., 2021). A retrospective study conducted by Michael also found a lack of evidence suggesting a correlation between the administration of perioperative dexamethasone during total joint arthroplasty and elevated levels of postoperative blood glucose (Nurok et al., 2017). The current evidence indicates that using dexamethasone during the perioperative period in arthroplasty procedures does not elevate the likelihood of experiencing wound infection or hyperglycemia (Allen et al., 2020; Vuorinen et al., 2019). In the present investigation, our findings indicate that there were no significant disparities in the occurrence of postoperative infection and hyperglycemia among the groups receiving repeat-dose dexamethasone or split-dose dexamethasone in comparison to those receiving single-dose dexamethasone. Nevertheless, it is important to exercise caution when administering intravenous dexamethasone to high-risk populations, such as individuals with diabetes mellitus and inflammatory arthritis, due to the substantial variability in dose regimens and inclusion criteria observed among the patients enrolled in the clinical trials.

Our study has several limitations. Firstly, heterogeneity arises inevitably from variations in dexamethasone dosage, perioperative pain management protocols, and the specific types (TKA and THA) included in the analysis. Secondly, the limited follow-up period precluded a reliable evaluation of the long-term efficacy and incidence of adverse events. A further limitation is potential publication bias, as the analysis depends on published literature; meanwhile, the lack of standardized pain assessment timepoints across trials hinders cross-study comparability. Lastly, demographic variability among patients complicates the generalization of findings to broader populations.

## Conclusion

Our findings indicate that the perioperative administration of repeat doses of dexamethasone is a viable and efficient strategy to reduce pain levels, shorten hospitalization durations, and lower instances of postoperative remedial analgesia compared with single-dose or split-dose dexamethasone administration. Importantly, these benefits were observed without any notable increase in adverse effects such as POVN, blood glucose abnormalities, and infections, which are commonly associated with the treatment.

## Data Availability

The original contributions presented in the study are included in the article/supplementary material, further inquiries can be directed to the corresponding authors.
